# Lay Health Workers experience of a tailored knowledge translation intervention to improve job skills and knowledge: a qualitative study in Zomba district Malawi

**DOI:** 10.1186/s12909-016-0580-x

**Published:** 2016-02-09

**Authors:** Lisa M. Puchalski Ritchie, Monique van Lettow, Jan Barnsley, Adrienne K. Chan, Michael J. Schull, Alexandra L. C. Martiniuk, Austine Makwakwa, Merrick Zwarenstein

**Affiliations:** Department of Medicine, University of Toronto, Toronto, ON Canada; Department of Emergency Medicine, University Health Network, Toronto, ON Canada; Institute of Health Policy, Management and Evaluation, University of Toronto, Toronto, ON Canada; Dignitas International, Zomba, Malawi; Dalla Lana School of Public Health, University of Toronto, Toronto, Canada; Dignitas International, Toronto, Canada; Sunnybrook Health Sciences Center, Toronto, ON Canada; George Institute for Global Health, Sydney, Australia; The University of Sydney, Sydney, Australia; National TB Control Program, Ministry of Health, Lilongwe, Malawi; Knowledge translation unit, Lung Institute, University of Cape Town, Cape Town, South Africa; Stellenbosch University Faculty of Health Sciences, Tygerberg, South Africa; Department of Family Medicine, Western University, London, ON Canada

**Keywords:** Lay health workers, Community health workers, Training, Knowledge translation, Educational outreach, Reminders

## Abstract

**Background:**

Like many sub-Saharan African countries, Malawi is facing a critical shortage of skilled healthcare workers. In response to this crisis, a formal cadre of lay health workers (LHW) has been established and now carries out several basic health care services, including outpatient TB care and adherence support. While ongoing training and supervision are recognized as essential to the effectiveness of LHW programs, information is lacking as to how these needs are best addressed. The objective of this qualitative study was to explore LHWs responses to a tailored knowledge translation intervention they received, designed to address a previously identified training and knowledge gap.

**Methods:**

Forty-five interviews were conducted with 36 healthcare workers. Fourteen to sixteen interviews were done at each of 3 evenly spaced time blocks over a one year period, with 6 individuals interviewed more than once to assess for change both within and across individuals overtime.

**Results:**

Reported benefits of the intervention included: increased TB, HIV, and job-specific knowledge; improved clinical skills; and increased confidence and satisfaction with their work. Suggestions for improvement were less consistent across participants, but included: increasing the duration of the training, changing to an off-site venue, providing stipends or refreshments as incentives, and adding HIV and drug dosing content.

**Conclusions:**

Despite the significant departure of the study intervention from the traditional approach to training employed in Malawi, the intervention was well received and highly valued by LHW participants. Given the relative low-cost and flexibility of the methods employed, this appears a promising approach to addressing the training needs of LHW programs, particularly in Low- and Middle-income countries where resources are most constrained.

**Electronic supplementary material:**

The online version of this article (doi:10.1186/s12909-016-0580-x) contains supplementary material, which is available to authorized users.

## Background

Low- and Middle-income countries (LMICs) and sub-Saharan Africa (SSA) in particular, have been hardest hit by the global shortage of skilled healthcare workers, with an estimated 1.5 million healthcare workers needed to meet the basic health care needs of the SSA population [1]. One strategy increasingly employed to address this shortage is to shift some health promotion and prevention tasks, and the delivery of simple treatments, to Lay Health Workers (LHW) [1, 2]. While shown to improve access to basic health services and positively impact a variety of health outcomes [2–4], lack of ongoing training and supervision are recognized barriers to LHW effectiveness [3]. Despite this awareness, information is lacking as to how LHW training and supervision needs may best be addressed, in order to maximize the quality and impacts of LHW contributions [3, 4].

Traditionally, ongoing training is provided offsite, with staff absent from the workplace for days to weeks, with significant associated actual and opportunity costs, and frequently with disruption of care delivery [2]. Given the resource constraints facing LMIC healthcare systems and lack of evidence for effectiveness of ongoing training as commonly delivered [2], efforts to develop and rigorously evaluate alternative, low-cost, training approaches are needed. While few studies have examined the use of knowledge translation (KT) strategies to improve health care outcomes in LMICs, KT strategies may be ideally suited to addressing the ongoing training needs of LHW programs, given their success and relative low cost.

Malawi has one of the most severe shortages of healthcare workers in SSA, with an average of 2.03 physicians and 36.8 nurses for every 100,000 population, in 2009 [5]. In response to this crisis, Malawi has significantly expanded its formal cadre of LHWs, termed health surveillance assistants (HSAs), and shifted a variety of basic health care services, including delivery of outpatient TB care for which they are primarily responsible, to this LHW cadre. Given the implications of the traditional approach to training with over 10,000 LHWs currently working in Malawi [5], alternative approaches are essential to ensuring that the continuing training needs of this important group of healthcare workers are met.

### Study context

In our recent study LHWs in Zomba district in Malawi identified lack of TB disease specific knowledge and clinical skills as the key barrier to their work as TB care providers [6], with many reporting a lack of both initial and ongoing training. Based on these findings, we developed and evaluated a KT intervention to address the identified training gaps. The intervention combined 2 KT strategies with proven success in changing provider behavior: educational outreach and reminders [7].

The educational outreach component involved a minimum of six training sessions, of 60–90 min each, held onsite during regular work hours over a 3 month period. Sessions were provided by participating health centres TB focus LHWs, who received training in the intervention itself and as peer trainers. As training of general LHWs is part of the TB focus LHWs regular duties, training occurred onsite during regular work hours, and payment of training stipends would limit the sustainability and scalability of the TB intervention and KT approach in future, neither peer trainers nor general LHW participants were paid stipends to attend or provide training. Sessions employed a combination of didactic and interactive techniques, and emphasized case-based role playing and discussion of challenging cases, to allow for efficient provision of TB specific knowledge and clinical and adherence counseling skills, and to provide opportunities for practice and exchange of ideas between participants. Topics included in the training included: TB transmission and natural history, the interaction of TB and HIV, TB treatment including side-effects and their management, common barriers to adherence, consequences of poor treatment adherence, and approaches to preventing and addressing poor adherence. The training manual and sessions were provided in English and employed the simplest language possible. The point-of-care tool was provided in Chichewa, to facilitate interaction with patients of relatively low literacy common among women, the elderly and in remote areas. Simplest language was defined by our local collaborators, rather than formal language testing, as in some cases a more complex term was felt to be commonly understood where a simpler term was not. Therefore, the specific language employed in the manual, training sessions and point-of-care tool, was chosen in consultation with local collaborators to reflect commonly recognized disease specific terms (ex. Germ rather than bacteria) and the tool pilot tested with LHWs at the central hospital. Clarification was provided from the study team and peer trainers in the local language Chichewa as needed. The point-of-care tool was designed to be referenced during patient interactions, with a guide to inquiring about and addressing adherence issues on one side, and pictorials to aid in patient education on the opposite side. Both the format and content of the training and point-of-care tool were initially based on the adherence and knowledge translation literature, then tailored to be context appropriate in consultation with the National TB unit and to address the training needs identified in the formative qualitative study [6].

The effectiveness of the intervention in improving TB treatment completion rates was evaluated in the cluster randomized controlled Trial, which showed a non-significant trend for effectiveness [8]. Pre-post knowledge testing was not conducted due to concerns that LHW fears about ‘testing’ would negatively impact participation in the training. The purpose of this qualitative study was to complement the quantitative evaluation of the intervention, by exploring the experiences and perceived changes in knowledge and clinical skill of LHWs who received the intervention, in order to understand what aspects of the intervention they found helpful, and to identify areas in need of improvement.

## Methods

### Design: qualitative study

#### Setting and participants

The study was conducted in Zomba district in southern Malawi (population 670,000, 80 % rural [9]), and utilized qualitative methods. Interviews were conducted with LHWs who had received the training. The intervention trial was conducted from April 2011 to March 2012. Ten to fifteen interviews were planned at each of 3 time blocks over the course of the 1 year intervention trial, with 3 participants to be interviewed at all 3 times, in order to assess for change across and within individuals over time.

Participants were selected utilizing mixed purposeful sampling [10]. A list of trained LHWs compiled by the peer trainers provided the initial sampling frame. Participants were initially randomly selected from the list of trained LHWS. We then purposively sampled participants from this list based on age, gender, and years of experience in the remainder of the first and subsequent rounds of interviews. At least one participant was selected from each of the 7 intervention sites, and a maximum of 3 from sites with a large number of trained LHWs. If a selected participant was not available, and not expected to become available during the time block, an alternate was selected.

During the initial round of interviews, participants were asked if they would be willing to be re-interviewed in both of the subsequent time blocks, and all agreed. In selecting participants to be re-interviewed, the goal was to select a subsample representing the range of initial responses. As there was little variation in LHWs perceptions in general and no systematic differences based on age, gender or years of experience during the first interview round (few concerns or negative perceptions), we selected participants to be re-interviewed in subsequent time blocks to represent the range of length and detail provided in the initial interviews. These criteria were utilized as we felt that shorter and less detailed interviews may represent a reluctance to provide negative feedback despite specific probing for constructive criticism. As experiences with the intervention may have varied by site, interviews were continued beyond saturation to ensure representation from all intervention sites in each time block.

Participants were recruited in person by a research assistant (RA) during field visits at each time block or by telephone when the LHWs selected was not available on-site. All approached to participate agreed. Written informed consent was obtained from all participants.

#### Ethics, consent and permissions

The study was approved by both the Malawi National Health Sciences Research Committee and the University of Toronto Research Ethics Board. Written informed consent was obtained from all participants. Data from this study will not be shared in keeping with the guarantee during the consent process that transcript data would be accessible only to the study team.

#### Data collection

Interviews were conducted face to face by two RAs, one male and one female, with all interviews in a given time block conducted by the same RA. Both RAs are experienced interviewers, native to Malawi, fluent in both English and Chichewa, and functioning at the level of a socio-linguistic interpreter [11]. All interviews were conducted using the same semi-structured interview guide (Additional file 1), with basic LHW characteristics collected at the start of each interaction. Interviews began with open-ended questions asking about participants’ experiences with the training and using the point-of-care tool, and moved toward progressively closed-ended questions as needed, to ensure all topics of interest were addressed. Topics of interest included: aspects of the training and tool felt to be helpful and not helpful, any concerns with the training or tool, and suggestions for improvement.

Interviews were conducted in a private area at/near the LHWs health centre. Sessions were conducted in the local language, Chichewa, audio recorded, transcribed verbatim, and translated by the RA who conducted the interview. A translation check was performed on 9 of 45 interviews (20 %), 2 early and 1 approximately mid-way through each round of interviews, by a second socio-linguistic translator to validate the quality and conceptual equivalence of the translations.

#### Analysis

Our approach to analysis was that of qualitative content analysis [12], with interviews as the unit of analysis in the context of the larger study aimed at exploring the experiences of LHWs with the intervention in order to understand the aspects of the intervention felt to be helpful and those in need of improvement. Transcripts were read and coded independently by 2 authors (LPR, MvL), looking for emergent themes and changes in participants perceptions overtime, both within individuals interviewed more than once, and across the participant groups over the 3 time blocks. NVivo 7 (QSR International Inc, Southport, UK) was utilized to organize and code the data into themes and subthemes. Discrepancies were resolved by discussion, and with input from the study RAs where provision of additional context and cultural perspective was needed to ensure clarity of interpretation of meaning.

## Results

### Characteristics of participants

Thirty-six healthcare workers participated in 45 interviews, with 14–16 interviews conducted at each time point, and 3 participants interviewed at all 3 time points, as planned. To ensure participation from all intervention sites at each time point, 2 participants were interviewed twice, as a result of a lack of LHWs available for interview during the final round. One additional participant was interviewed twice in error. All but 1 participant, a nurse, were general LHWs. All routinely provided care to TB patients. Although all were reported by the trainers to have finished the training, 3 acknowledged they had completed most but not all of the training at the time of their interview. Participants ranged from 26 to 57 years of age, with 2 to 20 years experience working with TB patients. Fifty-six percent of participants were male.

### Experience with the intervention as a whole

Without exception participants described the intervention as valuable. They reported improved confidence and ability to perform their role as TB care providers and adherence supporters, as a result of the knowledge and skills acquired and use of the point-of-care tool. While both components were highly valued, the point-of-care tool was noted to be particularly important, functioning both as a reference during patient interactions and as a general reminder/training refresher.

*“I have been working in TB services for four years but was just briefed on it and didn’t really know our role in TB care, but now the tool guides and reminds us in our work.” (LHW, 5 years TB experience)*

Details of the reported benefits, and suggestions for improvement, are outlined below. We found no systematic variation in themes or subthemes as a function of participant age, gender or years of experience. In addition, perceptions of neither the intervention as a whole nor its specific components were found to vary across the 3 time periods, either within or across individuals. No harms or detrimental effects of the intervention were reported.

### Reported benefits of the intervention

#### Increased knowledge

While the degree of improvement varied by participant, all reported having increased TB disease specific knowledge as a result of the training.

*“The education was like in service training, that has strengthened my weak areas and reminded me some forgotten areas” (LHW, 10 years TB experience)*

*“The training has been quite helpful to me since I have acquired a lot of new knowledge and it has also advanced my ability such that I am now able to offer services that I couldn’t before” (LHW, 6 years TB experience)*

Among the most commonly reported areas of improvement were understanding of the TB disease process and the interaction of TB and HIV.

*“I had little knowledge about TB, but through this education I am now more knowledgeable, particularly about the interaction between TB and the AIDS epidemic” (LHW, 3 years TB experience)*

Other frequently reported benefits of the training included: understanding the importance of treatment adherence and consequences of non-adherence, and increased ability to recognize and appropriately manage treatment side-effects.

*“Previously I could just issue the relevant drugs to clients and advise them to continue with medication since I had no knowledge to teach the client about the effects of adhering and not-adhering to treatment” (LHW, 11 years TB experience)*

*“The training was good especially issues to do with medication, since sometimes I used to meet clients who presented some complications and usually I could just regard such difficulties as normal. But through the training, I have learnt a big lesson, since I am now able to assess cases that require a clinicians attention and those that do not and advise the client accordingly” (LHW, 2 years TB experience)*

In addition to increased disease specific knowledge, participants reported gaining job-specific knowledge as a result of case-based discussions with their fellow LHWs. Reported job-specific knowledge gains included: increased awareness of the need for confidentiality, and improved understanding of job-specific tasks, including treatment documentation and medication dispensing.

*“I also learnt to keep everything I discuss with a patient confidential and be in doors to assure him of this” (LHW, 6 years TB experience)*

*“For example, it was difficult for me to issue drugs to patients correctly unless in the presence of a colleague, but I can now do it alone effectively after the training. I am able to calculate time periods at which a patient should come to collect drugs this was confusing to me before the training” (LHW, 5 years TB experience)*

*“One of the useful aspects is about documenting drugs in the registers. This has been a big problem here since we work in shifts at the work station, so information could be given and recorded wrongly. So this training has somehow helped us to follow one universal documentation of TB drugs dispensed” (LHW, 10 years TB experience)*

#### Improved clinical skills/benefits

Foremost among the reported clinical skills gained through the training, was an awareness of the importance of developing skills to fostering a positive patient-provider relationship.

*“I learnt how to openly interact with the patient. I didn’t learn this initially, hence hard for me to interact well with the patient, so he would leave for home unsatisfied. Now after being taught I have seen that patients are now free to speak their mind, telling us about issues affecting them” (LHW, 3 years TB experience)*

Other prominently reported clinical skills were an improved ability to provide patient education and an enhanced approach to patient follow-up, both in clinic and during tracing visits to the community.

*“It is helpful especially to us working in remote health centres where many people have low literacy level, so drawings on the chart aid some clients to grasp the information easily” (LHW, 2 years TB experience)*

*“Because we are now asking them questions, we are able to detect that this person is not taking medication properly and that is able to give us a way to help the patient” (LHW, 3 years TB experience)*

A final reported clinical benefit of the intervention was an increased confidence in their abilities, and personal satisfaction or a sense of pride in their work, as a result of appreciation expressed by patients and LHWs perceptions of improvements in their job performance.

“*The other thing that was helpful is the provision of the point-of-care tool, we are now able to know that this patient is here (stage in the treatment process) and that has empowered us, because when I am talking to the patient I am confident knowing that what I am telling them is not just what I have made up but it is something that is written to help sick people and that I think is a good thing.” (LHW, 3 years TB experience)*

*“(Since the training) I can see that there is some improvement in the way I discharge my duties as compared to previous times” (LHW, 4 years TB experience)*

### Suggestions for improvement

Several participants felt the training period should be extended, with one suggesting it be extended from 6 weekly sessions to run several months. A number suggested that the training would be better conducted in the traditional manner, with LHWs taken off-site for several days to a week for training, and provided with stipends and/or refreshments as incentives for training.

*“My concern is that the training period was short as we were having one session per week in order to give room to routine activities. It would be better if you organized a special venue and teach us there for one whole week.” (LHW, 18 years TB experience)*

*“The training was being conducted at the very (health) centre where we discharge our routine duties, hence subject to some disturbances. Our trainer was sometimes being interrupted, this was disturbing as the sessions were not running smoothly, hence it was sometimes difficult to follow.” (LHW, 3 years TB experience)*

*“Include training allowances to motivate participants since many HSAs (LHWs), admire their colleagues who receive a little something whenever they attend some training.”(LHW, 3 years TB experience)*

Others reported, the on-site training to work well, and indicated, that while stipends would be appreciated, they would prefer to have training without stipends, that to not have the training.

*“To me the training was conducted very well because our meeting hour was convenient most of the times in that there were no disturbances and even our trainer showed keen interest in teaching us.” (LHW, 4 years TB experience)*

*“The provision of stipends should not cause a worry to you. All I can request is that such trainings should be taking place regularly, because it is often times helpful to us.”(LHW, 6 years TB experience)*

Many participants suggested that quarterly refresher meetings would be helpful, with all sites reporting holding such meetings at least once per quarter. Some felt that refreshers should be conducted off-site with LHWs from multiple sites brought together.

*“I think it would also be helpful in our work if you organize regular joint meetings with fellow HSAs (LHWs) from other centres, so that together we can identify weak areas and also motivate one another.” (LHW, 5 years TB experience)*

Others felt, that refreshers should be conducted on-site in the same way the original training had been conducted.

*“Like it was done, I think if the same arrangement happens quarterly so as to refresh us after discharging our duties for a period of time. If it so happens that we encounter some obstacles in the process, we will bring that up during such meetings and gain new knowledge” (LHW, 16 years TB experience)*

A number of participants reported appreciation for the RAs visits to conduct interviews at the health centres, and suggested regular “support “visits.

*“Just to request you to keep visiting since your keen interest in the program makes us feel encouraged and motivated to work hard”(LHW, 12 years TB experience)*

While a number of minor additions were suggested for both the training and tool, only 2 were noted by several participants, increased coverage of HIV management and inclusion of a drug dosage reference.

*“It would be better if you combined both TB and HIV since there are many ailments associated with HIV, so we need to be guided on ways of taking care of co-infected patients” (LHW, 6 years TB experience)*

*“Add on the chart the aspect of dosage correspondent to age of a patient and the stage of treatment” (LHW, 5 years TB experience)*

Others specifically suggested that the tool not be changed for fear that it would become complicated and as a result less useful.

*“I can say that the tool is fine because if we add more it will just spoil it” (LHW, 3 years TB experience)*

## Discussion

To our knowledge few studies have employed and examined LHWs experiences with multi-component knowledge translation strategies tailored to address identified knowledge gaps. Many of the findings of the present study are in keeping with the few others conducted with similar techniques and in similar settings. Although designed for mid-level health workers, an early evaluation of the PALM PLUS intervention, also conducted in Zomba district Malawi and employing educational outreach and a clinical support tool, reported that participants felt empowered to provide better services as a result of the training and clinical support tool [13]. These findings are consistent with the reports of LHWs in the present study of increased knowledge and clinic skills allowing them to provide better care. In addition, some participants in the PALM PLUS study viewed the on-site approach favorably, indicating it saved travel time and costs, but many viewed lack of stipends as de-motivating [13]. Again, consistent with our findings of generally mixed views on the conduct of the intervention on-site and without stipends.

In addition, two studies with LHWs in LMICs found similar knowledge and skills gains, and improved clinical abilities reported by their LHW participants. Although not strictly educational outreach, Joseph et al. [14], report the experience of LHWs in Lesotho trained onsite through both didactic and hands-on training throughout the work day, with LHWs reporting gratitude for the knowledge and skills gained and for the impact they were able to have on patients. Finally, a study by Alcock et al. [15] with LHWs in India, who had received training offsite but with similar clinical skill development content to the present study and employed picture cards to assist with patient education, found positive changes among LHWs in terms of increased knowledge, confidence, and clinical skills, and found the pictures to be helpful in illustrating teaching points to patients.

Consistent with the generally positive views of LHWs to training and support tools to date, and that participants in the present study overwhelmingly perceived the intervention as helpful to them in their work, this appears to be a viable option for addressing LHW training needs and for providing one means of ongoing clinical support. In addition, the continued positive perceptions of the intervention over the course of the one year evaluation period may suggest a sustained benefit of the intervention and provide further support for the viability of the intervention for LHW TB training and for adaptation of the strategy to address LHW training needs in other health service areas. While some resistance was expected regarding the lack of incentives common to traditional training approaches, specifically stipends and removal from work to attend training, these issues were raised by a minority of participants. As such it may represent a less significant barrier to employing educational outreach methods than anticipated. In addition, the value of sharing lessons learned through group meetings may perhaps be addressed through other means such as telephone based peer mentorship networks that would allow for sharing of experience between peer trainers across sites, who can then pass along lessons learned to their local teams during regular staff meetings.

Given the urgent need for cost-efficient methods to address the training needs of LHWs, particularly in resource constrained settings, the KT approaches employed here, may be particularly well suited, considering their relative low-cost and flexibility. We feel however, that the substantial formative work conducted to understand the barriers and facilitators to LHWs providing optimal TB care in this context and efforts to tailor both the content and design of the intervention to the identified barriers and facilitators was critical to the success of the intervention. We would therefore recommend sufficient attention be placed on this formative phase.

There are several limitations to this study. While the RAs conducting the interviews, played no role in delivery of the intervention and specifically encouraged critical input, participants feeling supported by these visits may have been reluctant to provide negative feedback. Also, although TB policy is national, it is possible that not all findings will be generalizable to other districts of Malawi, as the study was conducted in Zomba District only. In addition, as the intervention was tailored to the local setting, direct adoption of the program by other TB programs, particularly outside Malawi, may not be appropriate. We do believe however, that the general approach employed with tailoring to local barriers and needs, is likely to have similar results. Language translation introduces the possibility of mistranslation of words or concepts, and may limit the understanding of the cultural context. However, we believe that the themes reported are accurately represented. Finally, as lack of training stipends was not specifically addressed in the interviews, it is possible that the findings in this regard may have been different if included as a specific interview question.

## Conclusion

Our findings suggest that a multi-component KT strategy, combining educational outreach and reminders, was well received and perceived as valuable by LHWs. In addition, lack of stipends and conduct of training on-site, proved a much less important issue than expected, and as such, may not represent a significant obstacle to use of this approach. Given these findings and the relative low-cost of the KT interventions utilized, if proven effective, the strategy employed here may well be a feasible option to addressing LHW training needs in Malawi, and perhaps more widely in LHW programs in other LMICs.

## Additional file

Additional file 1:
**Semi-Structured Interview Guide.** (PDF 63 kb)

## Abbreviations

HIV: human immunodeficiency virus
HSA: health surveillance assistant
KT: knowledge translation
LHW: lay health worker
LMIC: low- and middle-income countries
RA: research assistant
SSA: sub-Saharan Africa
TB: tuberculosis
