# Supplementary material for: Lay Health Workers experience of a tailored knowledge translation intervention to improve job skills and knowledge: a qualitative study in Zomba district Malawi
Source: BMC Med Educ. 2016 Feb 9;16:54. doi: 10.1186/s12909-016-0580-x (PMC4748491; doi:10.1186/s12909-016-0580-x)
Supplement: Additional file 1: — Semi-Structured Interview Guide. (PDF 63 kb) [file 12909_2016_580_MOESM1_ESM.pdf]

Additional file 1: Semi-Structured Interview Guide

1. *Do you have any questions?*

    If yes, answer questions.
    If no, may we proceed with the interview/focus group now then?

2. *First I would like to ask you a few questions about yourself.*

    Gender M/F
    Age ___
    Current Job HCW/TB focus HSA
    Location ZCH/Rural HC
    Years in current position ___
    Previous experience as HCW or TB focus HSA Y/N
    If Yes,
        location of previous experience ZCH/Rural HC/both
        total years of experience ___

3. *Have you completed the TB/HIV adherence training?*

    If yes, when? ____________
    If no, did you attend any session? Y/N
    If yes, how many? ___________
    If no, why not?_____________

* if they have not attended any sessions, they will be thanked for their time and the interview terminated.

4.Please tell me about your experiences with the TB/HIV adherence training?

5. What if any aspects of the training did you find useful, and how?

6. What if any aspects of the training did you not find useful, and why?

7. Do you use the point of care tool? If so, how often do you use it? What aspects of the point of care tool, do you find helpful and why?

8. What aspects of the point of care tool, do you not find helpful and why?

9. Do you have any suggestions for improving the training or tool, for example is there anything you feel should be added to or removed from the training or point of care tool?

10. Do you have any concerns with the training or tool?

11. Do you have anything you want to add or clarify?

12. Do you have any questions?

13. Thank you for your time.
